# Self-Guided Smartphone App (Vimbo) for the Reduction of Symptoms of Depression and Anxiety in South African Adults: Pilot Quantitative Single-Arm Study

**DOI:** 10.2196/54216

**Published:** 2025-01-30

**Authors:** Sherrie Steyn, Meggan Slabbert

**Affiliations:** 1 Vimbo Health SA (Pty) Ltd Sandton South Africa; 2 Private Practice Ballito South Africa

**Keywords:** treatment gap, mental health, health, depression, anxiety, South Africa, CBT, cognitive behavioral therapy, app-based intervention, mobile health, mental health app, smartphone, mobile phone

## Abstract

**Background:**

Barriers to mental health assessment and intervention have been well documented within South Africa, in both urban and rural settings. Internationally, evidence has emerged for the effectiveness of technology and, specifically, app-based mental health tools and interventions to help overcome some of these barriers. However, research on digital interventions specific to the South African context and mental health is limited.

**Objective:**

This pilot study investigated the feasibility of using an app (Vimbo) to treat symptoms of anxiety and depression in South African adults recruited from a community sample. The Vimbo app is a self-guided, cognitive behavioral therapy–based digital intervention for common mental health difficulties developed for the South African context.

**Methods:**

This pilot study used a naturalistic, single-arm design testing the Vimbo app over 12 weeks, from October 2020 to February 2021. Participants were recruited through the South African Depression and Anxiety Group and social media advertisements online. A 2-week retention period was used to allow for a minimum of 2 datasets. App usage and engagement metrics were extracted directly from the back end of the app. Based on the model, researchers expected many users to discontinue usage when their symptom levels entered a healthy range. Pre-post review of symptom levels was used to reflect on clinical recovery status at discontinuation after the retention period.

**Results:**

A total of 218 applicants met study eligibility criteria and were invited to download the Vimbo app. Of these, 52% (114/218) of the participants registered with the app, who indicated multiple variances of depression and anxiety symptoms ranging in severity from mild to severe. Two participants users withdrew from the study. Moreover, 69% (77/112) of users were retained, including 8 who had technical issues with their treatment. When comparing broad uptake across all interested participants, chi-square analysis indicated significantly reduced uptake in participants identifying as “unemployed but seeking employment” (*χ*^2^_4_=10.47; N=251; *P*=.03). When considering app usage for the entire cohort (n=69, excluding participants with technical issues), there was a mean of 72.87 (SD 71.425) total module pages read, a mean of 30% (SD 29.473%) of prescribed content completed, and a mean of 19.93 (SD 27.517) times engaging with tools and skills.

**Conclusions:**

Our findings support the case for continued exploration of app-based interventions for treating depression and anxiety in South Africa. Developing strategies to increase access and improve intervention uptake may prove essential to helping mobile health interventions make as significant an impact as possible. Future research should include a randomized controlled trial with a larger sample to further assess the efficacy of app-based interventions in treating mental health difficulties in South Africa.

## Introduction

South Africa currently has some of the poorest mental health outcomes globally, with 36% of the population feeling distressed or struggling [1]. Recent studies suggest that nationally, 25.7% (874/3402) of respondents reported scores ≥10 on the Patient Health Questionnaire-9 (PHQ-9) and 17.8% (606/3402) reported scores of >10 on the Generalized Anxiety Disorder-7 (GAD-7), indicating probable depression or anxiety [2]. Disorder prevalence varies widely by geographical province, which may reflect the variance in mental health resource availability and allocation [2,3]. These estimates are a marked increase from previous findings from the World Health Organization, which estimated that 4.6% of the population lived with a depressive disorder and 3.4% lived with an anxiety disorder [4]. This increase is part of a more significant global increase in cases of anxiety and major depressive disorders following the COVID-19 pandemic, including a 21.5% increase in the prevalence of these disorders in sub-Saharan Africa [5].

There is an ever-widening divide between increasing numbers of people experiencing mental health difficulties and access to adequate support or therapeutic intervention, known as the “treatment gap” [6,7]. Findings indicate that mental disorders often go untreated despite their high prevalence and associated impairment [6]. The treatment gap for depression and many anxiety disorders was more than 50%, increasing to as much as 80% in low- and middle-income countries for severe cases [6,8]. Government spending on mental health is below what is needed to adequately address this gap, with those in middle-income countries being the most affected [8].

South Africa is no exception. Despite having dedicated legislation, such as the Mental Health Care Act of 2002, there remains a significant treatment gap, especially in rural areas [9-11]. Recent studies suggest that for people in South Africa with mental health problems, only 1 in 10 receives the care they need [3]. This figure amounts to a staggering 92% treatment gap before the added burden of COVID-19. South Africa faces many barriers to bridging the treatment gap, including high levels of comorbidity and the relationship between common mental disorders (CMDs) and communicable diseases (such as HIV and tuberculosis), the apartheid legacy of institutional disparity, lack of adequate development of services, and a severe shortage of mental health professionals [9,11]. Research into cost-effective interventions is essential to inform policy decisions [9-11].

The use of technology and mental health apps in bridging barriers to this treatment gap is growing [7,9,12]. In 2018, approximately 99.5% of the population in South Africa had access to the 3G mobile network, and 85.7% had 4G/LTE coverage [13]. As of January 2020, South Africa had an estimated 3493 million active mobile web users [14].

The potential for app-based interventions to significantly reduce the cost of treatment, increase access in difficult-to-reach populations (such as rural communities), and reduce the need for clinician input and time is especially relevant to the South African context where these barriers have a more significant impact [6,9-11]. Several studies within South Africa have shown that mobile phones are already used as informal learning tools by patients and health care workers [15-20].

Despite this growing evidence and the number of mobile health (mHealth) apps, research is needed into apps designed specifically for the South African context and market. More generally, further research is needed to consider the cost-effectiveness and therapeutic efficacy of interventions for CMDs, especially mobile-based technology in South Africa [10,11].

Vimbo Health is a mental health assessment and treatment app, informed and developed based on a cognitive behavioral therapy (CBT) approach. The app streams users into courses for depression, anxiety and comorbid presentations using standardized psychometric measures, namely, the PHQ-9 and GAD-7. Subclinical users receive a well-being course focused on building awareness and resilience. CBT has proven effective in reducing symptom severity and functional impairment in both depressive and anxiety disorders [21-25].

This study aimed to investigate the feasibility of using the Vimbo app to reduce symptoms of anxiety and depression within an adult South African cohort. Using a single-arm, naturalistic design, we sought to pilot the app within a community sample as a self-guided intervention for those interested in supporting their mental health. We hypothesized that if offered a self-guided app (Vimbo), South African adults would voluntarily engage with the app to improve their mental health. We further hypothesized that Vimbo would reduce common symptoms of anxiety and depression symptom severity within 12 weeks of initial usage. Testing Vimbo’s safety and efficacy provides valuable knowledge about using app-based mental health interventions in South Africa.

## Methods

### Study Design and Procedure

This study used a naturalistic, single-arm design to assess the acceptability of a self-guided, digital intervention to reduce symptoms of depression and anxiety in South African adults, using multiple repeat measures administered in the app over 12 weeks.

Interested individuals provided consent (Multimedia Appendix 1) and completed a demographic questionnaire and PHQ-9 online via a web form. Eligible participants were invited to participate via email, offered technical support, and given 2 weeks to download and register with the app (February 1-15, 2021). App registration included completing the PHQ-9, GAD-7, and Work and Social Adjustment Scale (WSAS). Participants self-managed app engagement with psychometric measures administered biweekly in the app. Data were collected directly from the app over 12 weeks (ended May 10, 2021). At the study’s end, participants received a user experience questionnaire to collect qualitative feedback and were offered an opportunity to debrief with a researcher. Participants had 2 weeks to complete and submit the user experience questionnaire on the web. This study does not include these qualitative data, which will be reported separately.

### Ethical Considerations

This study received ethical clearance from the Human Sciences Research Council (HSRC) of South Africa on August 28, 2020. The HSRC Research Ethics Committee (REC) is registered with the South African National Health Research Ethics Council (REC-290808-015). The HSRC REC has the US Office for Human Research Protections Federalwide Assurance (organization 0000 6347). All participants signed a research study information and consent form via a web-based portal using a standardized form provided by the HSRC. Received consent forms were reviewed by researchers to ensure proper completion. All interested individuals were encouraged to contact the researchers should they have any question or wish to discuss their participation. Participation in the research was completely voluntary, with participants able to withdraw from the study at any point during the study period. The study was available to anyone comfortable communicating in English.

The Vimbo app is designed specifically for low data usage, both during download and regular use. The app has offline capability; as such, course content is downloaded once at the point of registration. Guided audio meditations are downloaded once when first played, so further playing does not consume any additional data. The app syncs to our back end when in daily use; however, expected use should not exceed 0.5 MB of data per day. The app is expected to use 126 MB over the 3-month study period. As such, participants were offered a once off reimbursement of R29.00 (approximately USD 2) to cover data usage based on local data costs.

Personally identifiable information stored in the mobile app back end was held on an encrypted Google cloud database, access to which was password protected and tightly controlled by the chief executive officer. Access to the back end is limited to only the chief executive officer and the software developer and restricted to critical use cases.

Data extracted from the back end were anonymized by removing or obfuscating personally identifiable information (name, email address, and phone number). Extracted data were kept in a password-protected and encrypted web-based folder hosted by Google with access granted only to the researchers. All electronic files generated by the research were maintained in the same electronic folders. Google Drive offers encryption and restricted access rights.

Informed consents and prescreening data were captured and stored electronically using the platform Jotform, which is password protected, is encrypted, and meets high security standards. Participants were anonymized through the use of coded identifiers, with any personally identifiable information including contact details stored in a separate, password-protected folder. Data were accessible only to the researchers and, due to necessity, the chief executive officer of Vimbo Health Ltd.

### Participants

Participants were recruited through the South African Depression and Anxiety Group and paid social media advertisements on Facebook from October 2020 to February 2021. Advertisements informed potential volunteers that the study assessed the effectiveness of an app for anxiety and depression over 12 weeks. Participation inclusion criteria included the following:

1. 18 years of age or older
2. South African residency
3. Access to an Android phone for the duration of the study
4. Comfortable using the app in English

Participants considered at risk of suicidality or self-harm as measured by a response of 2 and greater on question 9 of the PHQ-9 were excluded from participation in the intervention. Follow-up risk assessments and signposting were offered to these individuals.

**Figure 1:**
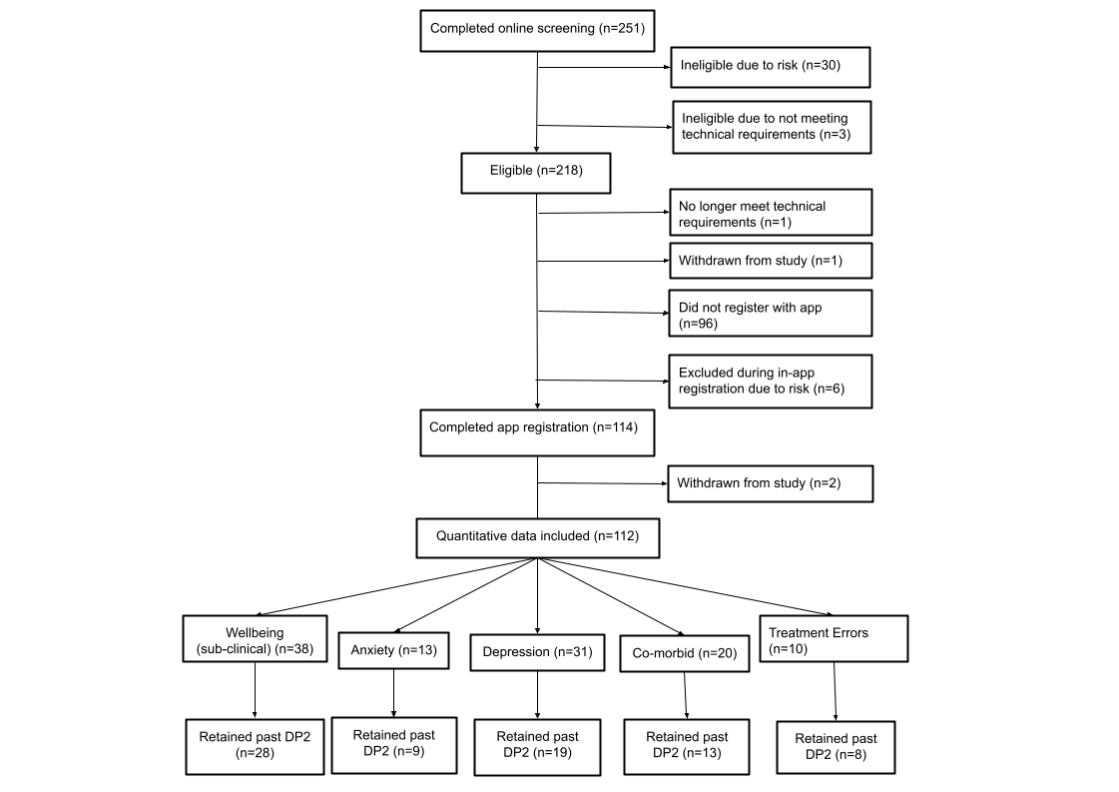
Flowchart of participants. DP2 is at 2 weeks after registration. DP2: data point 2.

A total of 4 participants were excluded based on technical criteria and 3 withdrew. An additional 36 were excluded due to risk concerns (PHQ-9 question 9 score of ≥2). Details of the flow of participants through recruitment and study are shown in Figure 1.

### Instrument and Procedure Overview

Interested persons were directed via a web-based link to a Jotform page for further study information and consent. Following digital receipt of study consent, individuals were asked to complete a demographic questionnaire and the PHQ-9 to help assess suitability for study participation. Following review of the consent form, demographic questionnaire, and PHQ-9 for inclusion and exclusion criteria, individuals were advised of their suitability for the study via their preferred communication mode.

Those participants who met the inclusion criteria and none of the exclusion criteria were invited to participate in the intervention and given 2 weeks to download and register with the app (February 1-15, 2021). Technical support was offered to participants to aid this process.

The registration process included the following:

1. Offering some brief information on the frequency of mental health problems and about the app, namely, that it is CBT based and aimed at assessing and treating mental health.
2. Entering their mobile number to get started. This number is used to text message a security code to authenticate the device.
3. Registering their details, including preferred name, email address, age, gender, and employment status.
4. Reading and agreeing to the terms and conditions.
5. Completing the PHQ-9 and receiving feedback on their score.
6. Completing the GAD-7 and receiving feedback on their score.
7. Completing the WSAS and receiving feedback on their score.
8. Completing a brief questionnaire on well-being.
9. Receiving feedback on the overall presentation.

After completing the registration process, participants self-managed their app engagement except for the psychometric measures, administered through pop-ups in the app every 2 weeks. Data were collected directly from the app over 12 weeks (ended May 10, 2021).

### Interventions

Following in-app registration, participants were placed into 1 of the following 6 treatment courses, based on their symptom profile:

1. Depression courses (PHQ-9 >10 or WSAS >20)
2. Anxiety course (GAD-7 >10)
3. Comorbid depression and anxiety course, addressing depression first (PHQ-9 and GAD-7 both >10)
4. Comorbid depression and anxiety course, addressing anxiety first (PHQ-9 and GAD-7 both >10)
5. Subclinical course focused on general well-being (for users who present with none to minimal symptom severity)
6. Low activity (subclinical scores, WSAS >20)

Users in the low activity group receive the same treatment course as those in the depression group.

Our courses are structured around CBT-based modules, which focus on skills development and encourage real-world practice. To support participants, we provide in-app tools and skills pages that facilitate the completion of suggested practice exercises, reflection, and skill integration. Measures are readministered fortnightly in the app. These allow the user to reflect on the impact their new skills are having on their symptom profiles. While no additional support is provided outside technical help, 21 random pop-up notifications are strategically used to encourage engagement. Details of course content and tools are included in Multimedia Appendix 2.

### Outcome Measures

App usage and engagement were measured by tracking interactions with the various aspects of the app, including content, skills, and tools. We also considered the percentage of overall content completed with the specific treatment course. Unfortunately, we could not reliably use the time stamp information to calculate the overall time spent on the app and individual session data due to possible inconsistencies in how these back-end data were collected. Participants were considered retained if they entered at least 2 complete sets of measures (PHQ-9, GAD-7, and WSAS), including those completed during registration.

In-app outcome measures allow the users to track their progress and symptom changes as a result of their efforts. These include the PHQ-9, GAD-7, and the WSAS [26-30].

1. PHQ-9: 9 items, 4-point Likert scale, measures depression severity (cutoffs: 5, 10, 15, and 20).
2. GAD-7: 7 items, 4-point Likert scale, measures anxiety severity (cutoffs: 5, 10, and 15).
3. WSAS: 5 items, 8-point Likert scale, measures functional impairment (cutoffs: 10 and 20).

Both the PHQ-9 and the GAD-7, widely used in research and clinical practice in health care and community settings, have shown reliable sensitivity within the general population [31,32]. These measures have also been tested for reliability in South Africa [33-36]. The WSAS, on the other hand, has proven to be a reliable and valid measure of impaired functioning, offering enough sensitivity to be an effective outcome measure [36,37]. All 3 measures were self-administered within the app, at registration, and every 2 weeks after that for the duration of the study, ensuring the credibility of our results. Reviewing pre-post changes on these measures allowed us to look at how clinical recovery may impact discontinuation with the app, with researchers theorizing that clinical recovery may coincide with discontinuation of the app in line with the underpinning theoretical model. Participants met clinical recovery if their final scores were below 10 on the GAD-7 and PHQ-9 after having initially scored above 10 on either measure.

### Statistical Analysis

All data analysis was conducted using IBM SPSS Statistics software (version 28; IBM Corp). Unless otherwise stated, the significance level for all tests was set to α=.05.

Demographic differences based on intervention uptake, retention, and recovery status were analyzed using chi-square and independent 2-tailed *t* tests. Frequency tables captured app usage and engagement. Unfortunately, due to technical issues, we could not accurately collate time-relative session data and, as such, could not reliably calculate the total number of usage sessions or the total amount of time spent on the app. Due to the small number of participants in the 2 comorbid streams, these were collapsed into a single group for analysis. Pre-post comparisons of symptoms levels on the PHQ-9 and GAD-7 allowed us to establish clinical recovery status. For these calculations, data collected during registration were compared with the last full set of primary measures completed in the app, regardless of the time point at which this was submitted (so entry and exit measures only). Therefore, any participants who completed only some of the primary measures would have their previous complete set of measures used for analysis.

## Results

### Participant Characteristics

A total of 218 people were invited to download and register the app. Moreover, 47% (104/218) of those invited did not register or no longer met criteria to participate in treatment. A total of 114 participants registered with the app; however, 2 withdrew from the study, leaving a total sample size of 112 participants.

The mean age of participants (n=112) was 35.21 (SD 9.6) years. The average PHQ-9 score was 9.58 (SD 6.02). In terms of ethnicity, 35% (39/112) identified as Black African, 34% (38/112) as White, 10% (11/112) as Coloured, 5% (6/112) as Indian, and 16% (18/112) as belonging to another ethnic group. The gender distribution was predominantly female, with 83% (90/112) identifying as female and 17% (21/112) as male.

Regarding primary language, 11% (12/112) of the participants spoke Afrikaans, 64% (72/112) spoke English, 11% (12/112) spoke isiZulu, and 14% (16/112) spoke another language. When asked about their current health condition, 43% (48/112) reported having a health condition, 53% (59/112) reported no health condition, and 5% (5/112) preferred not to say. Furthermore, 65% (31/48) of those who reported having a health condition reported a mental health issue. Employment status among the participants showed that 61% (69/112) were in some form of employment, 19% (21/112) were unemployed but seeking work, and 20% (22/112) fell into other categories.

When comparing broad uptake across all interested participants, chi-square analysis indicated significantly reduced uptake in participants identifying as “Unemployed but seeking employment” (*χ*^2^_4_=10.47; N=251; *P*=.03). Participants identifying with this employment category had the highest representation of those who did not register with the app (49/139, 35.3%). Also, those who categorized themselves as having a mental health diagnosis were significantly more likely to register (31/112, 28%) than those who did not identify as having a mental health diagnosis (81/112, 72%; *χ*^2^_1_=8.56; N=251; *P*=.003). No other significant demographic differences were found based on intervention uptake (all *P*>.05).

Moreover, 69% (77/112) of users were retained using a 2-week metric, with 31% (35/112) dropping out before completing a second set of measures in the app. Also, 45% (50/112) of users submitted a third set of measures, 29% (33/112) a fourth set, 22% (25/112) a fifth set, 12% (14/112) a sixth, and with only 5% (6/112) submitting a sixth set of measures at 12 weeks.

Table 1 looks at retention rates based on treatment group and symptom severity level. Moreover, 6% (2/35) of those participants who dropped out were missing treatment course data due to technical problems. Analysis of demographic information found no statistically significant differences associated with study retention (all *P*>.05). These percentages correspond to the overall proportionality in each treatment group.

Ten participants were missing treatment course information or had been placed in the incorrect treatment course due to technical error. Two (20%) of these 10 participants dropped out and 8 (80%) were retained. Of those who were retained, 7 (88%) of the 8 met the clinical symptom severity level criteria—neither of the participants who dropped out met the criteria for clinical symptom severity level (0/2, 0%).

**Table 1:** Comparison of treatment group allocation and symptom severity levels between retained and dropped out study participants.

Variable		Retained (n=77)	Dropped out (n=35)
**Participants (n=112), n (%)**		77 (69)	35 (31)
**Treatment group, n (%)**			
	Subclinical group	32 (42)	10 (29)
	Depression group	21 (27)	12 (34)
	Comorbid group	13 (17)	7 (20)
	Anxiety group	11 (14)	4 (11)
	Unknown due to technical error	N/A^a^	2 (6)
**Depressive symptom severity, n (%)**			
	Minimal	13 (17)	7 (20)
	Mild	25 (33)	7 (20)
	Moderate	21 (27)	17 (49)
	Moderately severe	12 (16)	4 (11)
	Severe	6 (8)	N/A
**Generalized anxiety disorder symptom severity, n (%)**			
	Minimal	17 (22)	9 (26)
	Mild	26 (34)	13 (37)
	Moderate	23 (30)	9 (26)
	Severe	11 (14)	4 (11)

^a^N/A: not applicable.

### App Usage

When considering overall app usage for the entire cohort (n=69, excluding participants with any technical issues), there was a mean of 72.87 (SD 71.425; range 0-278; mode 0) total module pages read, a mean of 3.36 (SD 2.169; range 0-10; mode 3) total modules accessed, and a mean of 19.93 (SD 27.517; range 0-126; mode 0) times engaging with tools and skills. Users (n=69) completed a mean of 30% (SD 29.473%; range 0%-100%; mode 0%) of prescribed content. Table 2 compares app usage and engagement across treatment groups.

There were no significant differences in app usage between those who met clinical recovery criteria and those who did not. Similarly, no significant correlations were found between changes in primary measures and app usage (all *P*>.05).

**Table 2:** App usage across treatment groups (total n=69, excluding users with technical errors).

Group		Module pages read (n)	Tools/skills usage (n)	Prescribed content completed (%)
**Depression group (n=19)**				
	Mean (SD)	69.68 (58.357)	19.05 (22.225)	29.40 (24.623)
	Range	210	86	89
	Mode	0	8^a^	0^a^
**Anxiety group (n=9)**				
	Mean (SD)	77.67 (84.109)	31.22 (37.181)	28.77 (31.151)
	Range	263	119	97
	Mode	0^a^	4	0^a^
**Comorbid group (n=13)**				
	Mean (SD)	66.85 (73.546)	12.54 (28.070)	20.07 (22.086)
	Range	260	104	78
	Mode	18^a^	3	5^a^
**Subclinical group (n=28)**				
	Mean (SD)	76.29 (77.676)	20.32 (27.356)	34.21 (34.832)
	Range	223	126	100
	Mode	0	0^a^	0

^a^Multiple modes exist. The smallest value is shown.

### Symptom Status at Discontinuation

The intervention follows a least intervention first approach, meaning that users are expected to engage with the app as needed until their symptoms return to a healthy level. Since CBT is time limited, the app focuses on real-world integration of the skills and techniques taught, with users expected to reduce engagement or discontinue use as their symptoms improve. Considering this, we have included a review of the recovery status of users based on their last complete set of measures submitted in the app, as an indicator of symptom status at point of discontinuation (last set of complete measures submitted in the app).

Of the 69 users who indicated clinically severe symptom levels at registration, 25% (17/69) met clinical recovery, 35% (24/69) did not meet clinical recovery, and 41% (28/69) did not have initial scores high enough to be considered before discontinuing use (last set of measures). Of those with clinical symptom levels at registration, 42% (17/41) met clinical recovery and 59% (24/41) did not meet clinical recovery based on their last submitted measures.

Moreover, 58% (11/19) of the depression course, 44% (4/9) of the GAD course, and 31% (4/13) of the comorbid course met recovery on the GAD-7 in their final scores. Also, 53% (10/19) of the depression course, 44% (4/9) of the GAD course, and 46% (6/13) of the comorbid course met recovery on the PHQ-9 in their final scores.

Based on current evidence, UK National Institute for Clinical Excellence guidelines recommend offering low-intensity CBT, including computerized CBT, for those individuals presenting with mild to moderate symptom levels and not for those individuals with moderate to severe symptom levels [38-41]. Considering this, Table 3 shows retention and clinical recovery status at discontinuation, when removing participants with severe symptom levels at registration.

**Table 3:** Retention and clinical recovery status at discontinuation or study end (excluding participants with severe symptom levels).

		Total participants, n	Dropped out, n (%)	Retained past DP2^a^, n (%)	Clinical recovery met, n (%)
**Clinical cohort**		51	20 (39)	31 (61)	16 (52)
	Depression group	30	12 (40)	18 (60)	9 (50)
	Anxiety group	4	1 (25)	3 (75)	3 (100)
	Comorbid group	17	7 (41)	10 (59)	4 (40)

^a^DP2: Data point 2.

## Discussion

### Principal Findings

This pilot study sought to test the feasibility of a self-guided, CBT-focused, mental health app–based intervention for South African adults who are experiencing mild to severe symptoms of depression and anxiety. Using an app specifically designed for the South African environment, we recruited 112 South African adults online and through support services, who were interested in using a mental health app. Participant engagement with the app was completely self-driven, with only technical support offered to users. Study retention rates and app usage indicate great potential for mHealth-based treatment; however, further technical development may be necessary to encourage greater engagement with content and tool use. In line with the treatment model, data suggest that most users who discontinued usage beyond the retention period did so after meeting clinical recovery. Although small, this pilot study is one of the first to test the usage case for an app-based intervention designed, developed for, and delivered within the South African context using a CBT model. Overall, these results support the continued exploration of using app-based interventions within the South African context to support the diagnosis and treatment of depression and anxiety.

In their survey of a low-income community in Johannesburg, van Rie and colleagues [42] found that 23% (283/1230) of the participants reported mild to severe depression. In our study, 27% (21/77) of the participants reported clinically significant depressive symptoms and 14% (11/77) reported clinically significant anxiety levels. Moreover, 17% (13/77) of the participants reported clinically significant depression and anxiety symptoms. Comparing our findings with a 2022 national survey where 25.7% (874/3402) of respondents reported PHQ-9 scores of 10 and greater and 17.8% (606/3402) reported scores of 10 and greater on the GAD-7 allows us to see better the impact the COVID-19 lockdown had on mental health [2].

Several authors have reflected on the disparity between individual enrollment and initiation in self-guided web-based interventions, with a systematic review finding that 33% to 88% of users who downloaded the app used it at least once [43-45]. Our uptake and retention rates are comfortably on the higher end of this scale and offer further evidence supporting the intervention. Other mental health app–based intervention studies report retention rates ranging from 5.5% to 70% after 30 days [46,47]. Our retention rates ranged from 69% at 2 weeks, 29% at 8 weeks, and 5% at 12 weeks. This steady reduction in retention is in line with the app model, which focuses on real-world integration of skills and learning with users expected to use the app for only as long as they need to reduce their symptoms to healthy levels. It further reflects the differing lengths of courses and individual progression through the intervention.

There was significantly reduced uptake in those identified as “unemployed but seeking employment.” This association may be due to issues with communication during the recruitment and app registration process, especially since subsequent analysis found no significant relationship between employment and retention. Several mHealth studies have found reduced retention in those who identified as unemployed [46,48]. The significantly increased uptake in those participants who reported having a mental health diagnosis suggests that already having a mental health diagnosis may act as a motivator for seeking support and engaging in research.

We found that users in a clinical course completed an average of 26% of their prescribed content, equating to approximately 2.1 modules completed per user. In a similar study, the participants completed an average of 2.6 (SD 2.7) modules. However, this included some access to coaching [49]. Aizenstros et al [46], in their study of a smartphone-delivered behavioral activation intervention, found that users completed an average of 5.46 (range 1-65; n=238) app-based activities over 30 days. Their findings were comparable with those of Watts et al [50], whose study participants’ mean aggregated score for homework completion within the mobile-based intervention was 11 (range 6-17; n=35).

Although usage is comparable, if not higher than other app-based interventions, numerous studies suggest that outcomes may be further improved if a mental health care professional guides the digital intervention [51-53]. Anton et al [49] found increased treatment outcomes and engagement in participants who completed at least 4 modules compared with those who completed fewer, suggesting a minimal dosage effect. However, it is tricky to know since interventions may not be directly comparable in amount and length of content [49]. However, in a similar small-scale pilot, Moghimi et al [54] found significant improvements in the GAD-7 and PHQ-9 for participants of their 9-week mHealth intervention. Their study had better overall retention, suggesting room for improving intervention efficacy through increased treatment adherence. Considering the scalability and impact on accessibility that mHealth interventions offer, even small gains can lead to considerable public health gains [23]. As other authors have noted, this is especially relevant to vulnerable populations, including those who may be geographically isolated and those with limited access to suitable resources [19,55].

As mentioned, there is a significant treatment gap related to the assessment and treatment of mental health disorders, specifically those individuals with a comorbid diagnosis, that is, evidenced by 2 or more mental health difficulties or disorders experienced simultaneously. The applicability of mHealth for people accessing mental health services and the possibility of reducing hospital admissions and improving symptom severity, engagement, and treatment are undeniable for clinical practice. Furthermore, this applicability extends to using mHealth as a stand-alone process or a process used in conjunction with other mental health care interventions, such as individual and group therapy or support groups and psychiatric interventions. The potential for app-based interventions to significantly reduce the cost of treatment, increase access in difficult-to-reach populations (such as low-income or rural communities), and reduce the need for clinician input and time is especially relevant to the South African context where these barriers have a more significant impact [6,9-11,19,56].

The app allows users to track their symptom progression easily, is designed for low data usage, and includes offline functionality. Furthermore, the app can be accessed using a voucher code and text message verification, allowing almost immediate access without professional guidance. This feature increases immediate access to treatment and reduces barriers such as wait times, travel and transport difficulties, and stigma. This is especially noteworthy in light of the findings by Forman-Hoffman et al [57], whose investigation found no significant differences between participants in underserved rural or metropolitan areas regarding usage, satisfaction, and treatment outcomes.

The Vimbo app has multiple usage cases, including as a stand-alone intervention or as a therapeutic tool and resource. This adaptability allows it to be integrated at various care levels within a stepped-care system to effectively create easily personalized intervention options into current health systems [12,23]. Using this stepped-care implementation directly into current health systems encourages a reduced caseload for care providers and better use and distribution of resources [7,10]. Furthermore, research suggests that using a blended CBT model that integrates a digital program may improve working alliance and outcomes, suggesting that treatment as usual may benefit by including access to the Vimbo app [58].

### Strengths and Limitations

This pilot study possesses several strengths. One of the primary strengths is its contextual relevance, as it is among the first to explore app-based digital interventions within the South African context. This offers valuable insights into the scalability of such interventions to larger settings and services. The study’s naturalistic setting, community orientation, and diverse participant age range offer realistic and generalizable insights into everyday usage and engagement. The research points to future digital intervention developments for rural and urban South Africa and has provided Vimbo Health with data for app improvements.

However, it is important to note the study’s limitations, which could impact the generalizability of the findings. While qualitative data on user experiences were collected, they were not included in this report due to the significant amount of quantitative data, with plans to publish these findings separately. The small sample size limits the statistical power of the study, making it challenging to extend the findings broadly. The context of the pandemic may have influenced user registration, engagement, and outcomes, indicating a need for further research in nonpandemic conditions. The reliance on self-reported data could introduce biases and affect the accuracy of the results. The app’s availability only in English excluded non-English speakers. Future iterations should consider localizing the app to include all South African languages.

### Conclusions

This pilot study takes the first critical steps to establish the feasibility of using self-guided, app-based treatment for anxiety and depression to help address the substantial mental health treatment gap in South Africa. The 69% (77/112) retention of users for a minimum of 2 weeks shows that mHealth interventions are a viable treatment modality, acceptable to the South African community. However, only 52% (114/218) of individuals invited to participate in the research registered with the app, suggesting the potential for greater impact if the access and onboarding process can be refined and improved to increase uptake. Overall, our results show promise for the use of digital technologies in addressing symptoms of anxiety and depression in South African adults struggling with their mental health. However, due to the pilot nature of this study and the limited number of other studies in this field within South Africa that have examined the use of digital interventions, further studies are needed to better understand treatment parameters including efficacy and dosage response. Large-scale, longitudinal studies are necessary to better assess the impact digital interventions have on mental health symptoms. Findings from this initial study offer support for app-based psychological interventions to bridge the ever-widening treatment gap.

## Appendix

Multimedia Appendix 1 Participant information sheet and consent form.

Multimedia Appendix 2 Vimbo version 1 app outline.

## Abbreviations

CBT: cognitive behavioral therapy
CMD: common mental disorder
GAD-7: Generalized Anxiety Disorder Assessment-7
HSRC: Human Sciences Research Council
mHealth: mobile health
PHQ-9: Patient Health Questionnaire-9
REC: Research Ethics Committee
WSAS: Work and Social Adjustment Scale

## Data Availability

All data generated or analyzed during this study are included in Multimedia Appendices 1-2.
