# Supplementary material for: Self-Guided Smartphone App (Vimbo) for the Reduction of Symptoms of Depression and Anxiety in South African Adults: Pilot Quantitative Single-Arm Study
Source: JMIR Form Res. 2025 Jan 30;9:e54216. doi: 10.2196/54216 (PMC11826938; doi:10.2196/54216)
Supplement: Multimedia Appendix 1 [file formative_v9i1e54216_app1.pdf]

PARTICIPANT INFORMATION SHEET AND CONSENT FORM

Vimbo: A Cognitive Behavioural Therapy based self-help mobile application for reducing symptoms of common mental health difficulties and increasing subjective well-being.

Who we are

Hello, we are Researchers, Entrepreneurs and Psychologists that are interested in improving mental health and well-being via online interventions. Specifically looking at Vimbo, a mobile application developed by us for you.

What we are doing

We are conducting research on a Cognitive Behavioural Therapy based self-help mobile application called Vimbo, specifically looking at how Vimbo may affect people who are experiencing mental health difficulties. We would also like to see how it affects well-being.

Your participation

We are asking you whether you will be willing to be a part of this study that involves the following two-stage processes:

- Use and interact with the Vimbo application by completing the full course content and modules. This will include engaging with the suggested practice and in-app skills and tools daily, as well as fortnightly questionnaires.

  We expect daily activities to take approximately 5-30 minutes of participation, spread across using tools, reading content, and practising skills. Fortnightly, you will also be requested to complete 4 psychological measures questionnaires taking approximately 10-15 minutes in total to conduct.

  The fortnightly questionnaires will help track your progress and allow you to reflect on which techniques work or don't work for you. You may take a break between questionnaires if you need to - each individual questionnaire is expected to take approximately 2-5 minutes to complete

- After completion of the 12 week period, to complete the Vimbo User Experience Feedback Questionnaire, which you will be given 2 weeks to complete and submit/return via email or an online portal.

You will need access to an Android Smartphone for the duration of this period. Unfortunately, we have not yet developed the platform for Apple Smartphones.

Please understand that **your participation is voluntary,** and you are not being forced to take part in this study. The choice of whether to participate or not is yours alone. If you choose not to take part, you will not be affected in any way whatsoever. If you agree to participate, you may stop

participating in the research at any time and tell us that you don't want to continue. If you do this, there will be no penalties and you will not be prejudiced in any way.

Expected Impact on Your Time

We expect daily activities as part of the programme to take approximately 5-30 minutes of participation, spread across using tools, reading content, and practising skills. Every two weeks, you will also be requested to complete a questionnaire taking approximately 10-15 minutes to conduct.

Confidentiality

All identifying information will be kept in a password protected and encrypted online folder and will not be available to others and will be kept confidential to the extent possible by law. The records from your participation may be reviewed by people responsible for making sure that research is done properly, including members of the ethics committee at the Human Sciences Research Council. (All of these people are required to keep your identity confidential.) Otherwise, records that identify you will be available only to people working on the study, unless you give permission for other people to see the records.

Your answers will be stored electronically in a secure environment and used for research or academic purposes now or at a later date in ways that will not reveal who you are. All future use of the stored data will be subject to further Research Ethics Committee review and approval.

We will not record your name anywhere and no one will be able to connect you to the answers you give. Your answers will be linked to a fictitious code number or a pseudonym (another name) and we will refer to you in this way in the data, any publication, report or other research output.

Risks/discomforts

Those participants who appear to be experiencing risk, as measured by a change in their scores in the in-app questionnaires, will be contacted by the researchers and offered further support, where this is deemed necessary by the researchers for the welfare and safety of the participant. In agreeing to participate you agree to this contact.

Benefits

There are no immediate benefits to you from participating in this study. However, this study will be useful to us in that we hope it will promote understanding of Vimbo: A Cognitive Behavioural Therapy based self-help mobile application. You will also be offered free access to the app for 6 months.

If you would like to receive feedback on our study, we will record your phone number on a separate sheet of paper and can send you the results of the study when it is completed sometime after.

Reimbursement of Personal Data Usage

We recognise that you will be using your own mobile data to access the platform. The Vimbo App is designed specifically for low data usage, both during download and regular use. The app

mostly functions offline as the content is downloaded once as part of the in-app registration process, and guided audio meditations also only need to be downloaded once. We estimate that over the 3 month study period, you will utilise approximately 126MB of personal data. Thus we will reimburse you for this in cash based on the current cost of 150MB of data, which equates to R29 based on current Vodafone, MTN, Cell C, and Telkom rates. It will be paid once-off at study commencement via an Electronic Funds Transfer directly into your bank account, or by other means to be arranged with you should you not have a bank account (e.g. a compensatory mobile data purchase).

Who to contact if you have been harmed or have any concerns
This research has been approved by the HSRC Research Ethics Committee (REC). If you have any complaints about ethical aspects of the research or feel that you have been harmed in any way by participating in this study, please call the HSRC's toll-free ethics hotline 0800 212 123 (when phoned from a landline from within South Africa) or contact the Human Sciences Research Council REC Administrator, on Tel 012 302 2012 or
e-mail research.ethics@hsrc.ac.za.

If you have concerns or questions about the research, you may contact the project leader
Sherrie Steyn via email:
research@vimbohealth.com
and/or
Co-Researcher Meggan Slabbert via telephone or WhatsApp:
076 280 3016

For technical support related to the application please do not hesitate to contact Tafi Mazikana our CEO on:
support@vimbohealth.com
0638616780

CONSENT
I hereby agree to participate in research on **Vimbo: A Cognitive Behavioural Therapy based self-help mobile application for reducing symptoms of common mental health difficulties and increasing subjective well-being**. I understand that I am participating freely and without being forced in any way to do so. I also understand that I can stop participating at any point should I not want to continue and that this decision will not in any way affect me negatively. I understand that this is a research project whose purpose is not necessarily to benefit me personally in the immediate or short term. I understand that my participation will remain confidential.

Signature of participant:…………………………….. **Date**:…………………..

I understand that the information that I provide will be stored electronically and will be used for research purposes now or at a later stage.

Signature of participant:…………………………….. **Date**:…………………..
