# Supplementary material for: Self-Guided Smartphone App (Vimbo) for the Reduction of Symptoms of Depression and Anxiety in South African Adults: Pilot Quantitative Single-Arm Study
Source: JMIR Form Res. 2025 Jan 30;9:e54216. doi: 10.2196/54216 (PMC11826938; doi:10.2196/54216)

Vimbo Version 1 App Outline

Registration

The app registration process includes:

- Enter their mobile number to get started. This number is used to SMS a security code to authenticate the device.
- Register their details, including preferred name, email address, age, gender and employment status.
- Reading and agreeing to the Terms and Conditions
- Completing the Patient Health Questionnaire 9 and receiving feedback on their score
- Completing the Generalised Anxiety Disorder 7 and receiving feedback on their score
- Completing the Work and Social Adjustment Scale and receiving feedback on their score
- Completing a brief questionnaire reflecting on general wellbeing (5 questions)
- Receiving feedback on the overall presentation

After completing the registration process, participants self-managed their app engagement except for the psychometric measures (PHQ-9, GAD-7, WSAS) and 5 questions about general wellbeing, which are administered through pop-up in-app every two weeks.

Treatment Groups

Scores from the psychometric measures administered at registration are used to stream the app user into one of the following treatment groups:

- Depression (PHQ-9 T>=10; GAD-7 T<10)
- Anxiety (PHQ-9 T<10; GAD-7 T>=10)
- Comorbid Depression and Anxiety- low activity start (PHQ-9 T>=10; GAD-7 T>=10)
- Comorbid Depression and Anxiety- worry start (PHQ-9 T>=10; GAD-7 T>=10)
- Wellbeing (PHQ-9 & GAD-7 >10.)
- Low Activity (sub-clinical scores, WSAS T>20)

Users in the Low Activity Group receive the same treatment course as those in the Depression group. Users who had comorbid Depression and Anxiety were administered the additional question below to decide initial treatment focus:

Although Vimbo will help you with both, it is helpful to decide on one to focus on first. Below are two statements- choose the one which feels most important TO YOU at the moment:

A. “I just can’t seem to get anything done. I’m always tired even though I spend more time in bed. I’ve started avoiding certain things because I just don’t feel up to it. I can’t remember the last time I had fun.’

B. ‘I feel like my mind never stops! I’m constantly worrying about everything and my body feels stressed and tense. I’m struggling to focus and as a result, am finding it difficult to cope with my day to day life.’

Answer A gets streamed into Comorbid Depression and Anxiety- low activity start.

Answer B gets streamed into Comorbid Depression and Anxiety- worry start .

Users considered high risk based on a response of >2 to Q9 on PHQ-9 were given the following feedback in-app:

In your answers, you mentioned thinking about being better off dead or of hurting yourself daily. Although many of us have these types of thoughts when we are struggling with our mental health, they can be incredibly difficult to manage and may result in harm or even death.

Considering how difficult things are for you right now, we would not recommend using Vimbo right now. Since Vimbo is an app-based approach we cannot offer you the 1-1 support you likely need right now. We recommend speaking to a Doctor or Mental Health professional personally to find a more suitable approach for your current difficulties.

Please reach out to someone, call emergency services or visit your nearest hospital to get immediate help if you feel you may hurt yourself or try to take your life. Your life does matter!

These users were contacted for follow-up risk assessment and support as part of the research protocols.

Content

Content is broken down into modules covering specific CBT skills and techniques. Below is an overview of what each module covers to which group it is administered:

1. Introduction: covers psychoeducation about Cognitive Behavioural Therapy, app usage and functionality, S.M.A.R.T Goal setting and creating a Problem Statement Summary (All groups).
2. Relapse Prevention (All groups)
3. Psychoeducation on symptoms and the 5 areas model of depression (Depression and both Comorbid groups)
4. Behavioural Activation (Depression and both Comorbid groups)

5. Unhelpful Thinking Styles and Thought Challenging (All groups)
6. Problem-solving (All groups)
7. Basic Mindfulness (All groups)
8. Psychoeducation on symptoms and the 5 areas model of anxiety (Anxiety and both Comorbid groups)
9. Worry Management (Anxiety and both Comorbid groups)
10. Active Relaxation (Anxiety, Wellbeing and both Comorbid groups)

Tools

Interactive:

- Mood Monitor and Daily Journal- reflect on mood, diet, sleep, activity level, thoughts on what went well and gratitude. Tracks previous entries for review
- 5 Areas Model- allows users to review triggering situations using the 5 areas model reviewing thoughts, emotions, behaviours, physical sensations and environmental triggers. Tracks previous entries for review.
- Urgent Support and Crisis Signposting

Audio:

- Progressive Muscle Relaxation (PMR) Practise
- Visualisation Exercise
- Short Breathing Exercise
- Mindfulness of Emotions
- Mindfulness Breathing

Skills: Content-based worksheets reviewing rationale and practise steps

- SMART Goal Setting
- Behavioural activation, including activity list, ranking and planner.
- Worry Management Techniques
- Problem Solving
- Thought Challenging
- Active Relaxation techniques including PMR, controlled breathing and visualisation.
- Mindfulness exercises including Mindful Breathing, Mindfulness of Emotion, Mindful Eating, Mindfully Brushing Teeth, 5 sense exercise, Regulating Attention Exercise and Body Scan Exercise

Images of App

Main Menu:

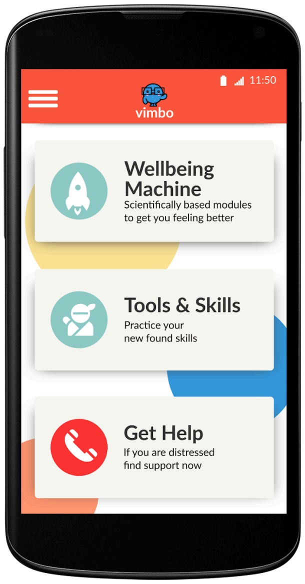

Module Content Menu:

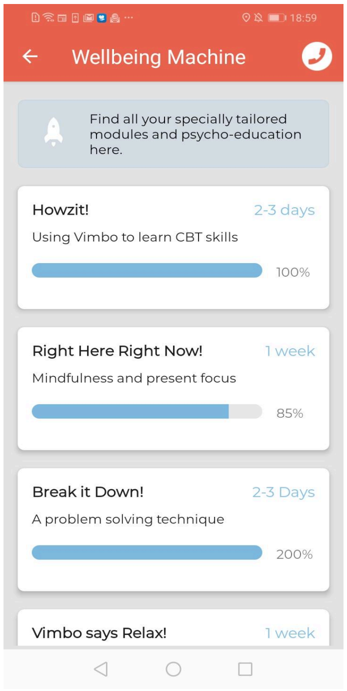

Example of Module Content:

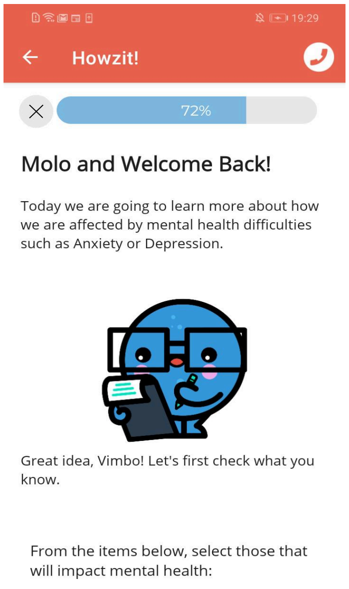

Example of Interactive Tool:

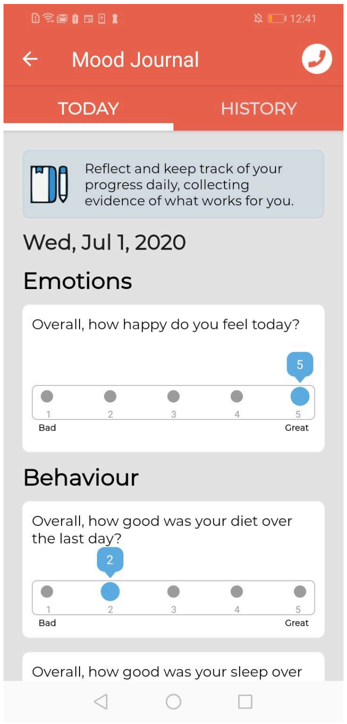

Supplement: Multimedia Appendix 2 [file formative_v9i1e54216_app2.pdf]
